# Nonoperative Treatment of Midshaft Clavicle Fractures in Adults

**DOI:** 10.2174/1874325001812010001

**Published:** 2018-01-17

**Authors:** Sören Waldmann, Emanuel Benninger, Christoph Meier

**Affiliations:** Clinic for Orthopaedics and Traumatology, Department of Surgery, Kantonsspital Winterthur, Brauerstrasse 15, CH-8401, Winterthur, Basel, Switzerland

**Keywords:** Clavicle fractures, Midshaft, Adult, Treatment, Conservative, Adults

## Abstract

Clavicle fractures are among the most common skeletal injuries accounting for 2-5% of all adult fractures. Historically, nonoperative treatment of midshaft clavicular fractures was considered the gold standard of care. Furthermore, nonoperative treatment has been challenged by an increasing popularity and rate of surgical fixations in recent years despite a lack of clear evidence in the current literature. Most fractures are suitable for conservative treatment. There is solid evidence in favour of nonoperative treatment for fractures with a displacement of less than 2cm and remaining contact of the bone fragments. Clear indications for conservative treatment *versus* surgical fixation of displaced midshaft fractures have not finally been established yet, leaving some questions and problems unanswered. Furthermore, there are no evidence-based recommendations concerning the kind and duration of shoulder immobilisation with no clear advantage for any treatment modality.

## 1 INTRODUCTION

Clavicle fractures are among the most common skeletal injuries accounting for 2-5% of all adult fractures with an incidence of 29-64 cases per 100.000 [1, 2]. These injuries often result from moderate to high-energy mechanisms such as sports injuries or road traffic accidents. Sports injuries are responsible for nearly half of all clavicle fractures. This group includes in particular young high-demanding male individuals, whereas low-energy fractures in elderly people predominantly result from falls. Pathological fractures caused by metastatic or metabolic disease are rarely seen [3].

Historically, nonoperative treatment of midshaft clavicular fractures was considered the gold standard of care. This recommendation is based on the analysis of 2000 patients with a very low non-union rate of 0.13%, reported by Neer in 1960 and Rowe’s publication from 1968 with an observed nonunion rate of 0.8% in 566 midshaft clavicular fractures [4, 5]. However, there has been no uniform conservative treatment modality yet and different conservative interventions are commonly applied. Furthermore, nonoperative treatment has been challenged by an increasing popularity and rate of surgical fixations in recent years despite a lack of clear evidence in the current literature [6] Fig. (**1A** and **B**).

### 1.1 Fracture Localization and Biomechanics

Most clavicular fractures occur in the mid-part of the clavicle (80%), about 12-15% are laterally localized and only a few fractures affect the medial part of the bone [5, 7, 8].

**Fig. (2A-F):**
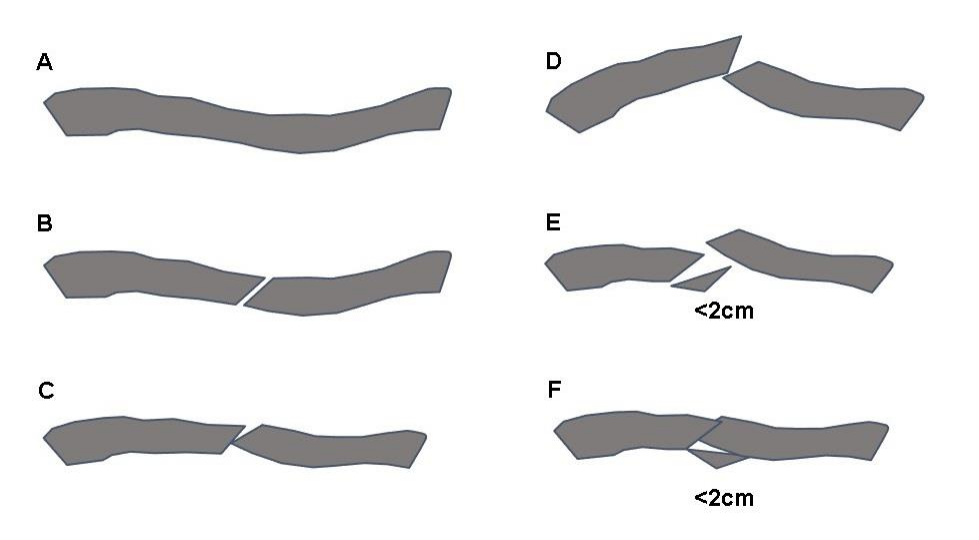
Nonoperative treatment advised A. Incomplete B. Alignment C. Minimal displacement D. Dislocated with contact E. Displaced with distance 2cm F. Minor Shortening.

The trauma mechanism of clavicle fractures is typically induced by a direct blow to the shoulder, rather than by a fall on the outstretched hand [9]. The clavicle is an S-shaped relatively thin bone with a larger diameter in the medial part and a strong ligamentous fixation at its distal end. The midshaft is susceptible to fracture where there are no strong ligaments, muscle coverage is absent and the curved bone is weaker. These fractures are usually complete and show an either oblique or transverse, often multifragmentary fracture pattern. In 73%, midshaft fractures are displaced without any contact of the bone fragments [2] (Fig. **2A**-**F**).

### 1.2 Indications for No Operative Treatment

**Fig. (3A-E):**
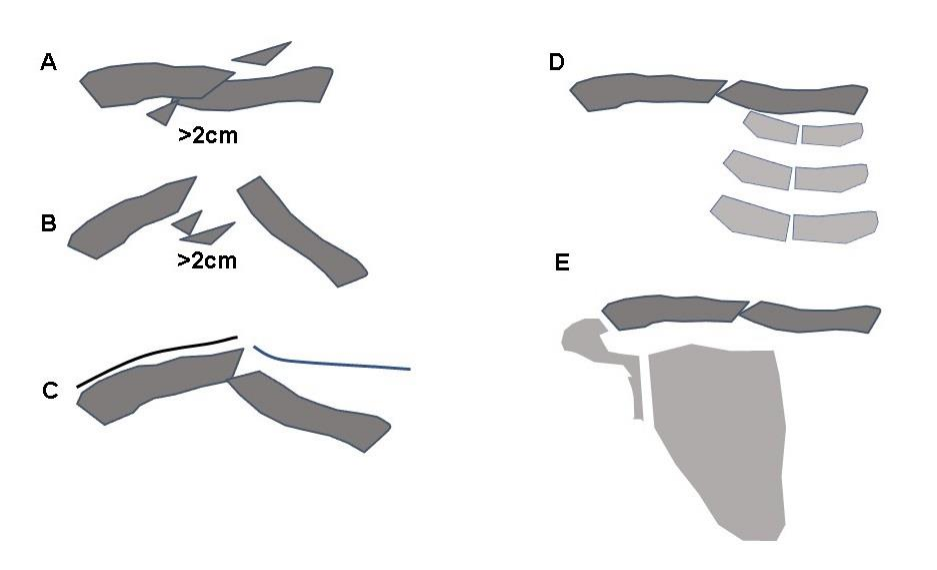
Operative treatment advised A. Shortening >2cm B. Displaced without contact >2cm C. Skin lession D. Combination with ipsilateral serial rib fractures E. Floating shoulder

The primary goal of treatment is to restore shoulder function to a normal level by setting preconditions which allow the clavicle to heal with minimal deformity, no loss of shoulder motion and minimized pain [10]. There is no controversy that undisplaced fractures and fractures with cortical alignment are successfully treated by conservative measures [2]. Nonoperative treatment is also recommended for fractures with a displacement and shortening of less than 2cm [11]. Good shoulder function equivocal to operatively treated fractures can be achieved with a low nonunion rate. However, surgery should be considered for open fractures, compromised skin conditions, neurological deficiencies, vascular injury, ipsilateral serial rib fractures or floating shoulder [12]. Considering these indications, more than 50% of all midshaft fractures of the clavicle in adults are suitable for nonoperative treatment with excellent functional outcome [13] (Fig. **3A**-**E**).

The best treatment for fractures with a displacement and shortening of more than 2 cm is still controversially discussed in the literature [14-16]. There is some good evidence that the nonunion rate is significantly higher when these fractures are managed nonoperatively (0-34%) compared to surgical treatment (0-3%) [17]. In a meta-analysis of randomized clinical trials investigating conservative treatment *versus* surgical care for displaced midshaft fractures, McKee et al reported an overall nonunion rate of 15% *versus* 1% of all included studies [17]. However, the functional outcome of healed fractures is similar in both groups and the better outcome of surgical fixation appears to result mainly from the prevention of nonunions [18]. Two number-to-treat analyses demonstrated that more than 5-6 patients have to undergo primary surgery in order to prevent one single nonunion [18, 19]. The authors could demonstrate that plate fixation increases the union rate significantly for displaced fractures but they found no difference concerning the functional outcome of the shoulder with similar Constant-Murley and DASH scores at all time points [19].

In a systematic review, all reported predictors associated with nonunion following nonoperative treatment of displaced midshaft clavicular fractures were analyzed [20]. Displacement was found to be the most likely predictor. Smoking, fracture comminution, shortening, advancing age and female gender were identified to be doubtful risk factors whereas fracture angulation, a vertical fragment, the presence of associated injuries, and other factors did not demonstrate any impact on the development of a nonunion [20].

Patient satisfaction regarding the cosmetic result is reported to be higher after surgical treatment [18, 21]. Pain control during the first 5-6 weeks after trauma is also more efficient after osteosynthesis compared to nonoperative treatment [7, 22, 23]. Conservative fracture management may also be associated with a longer time of incapacity to work compared to surgical fracture care [24]. However, Robinson *et al*. could not show any difference regarding return to work, not even for manual work [4]. Neither the number of patients returning to their sport nor the timing of the return to sport differed between conservative treatment and plate fixation [4]. From an economic point of view, the overall cost of treatment is significantly higher for plate fixation than for the nonoperative management despite the much lower rate of nonunion [4, 18].

Newest literature shows still no association of shortening and functional outcome or patient satisfaction in healed fractures, but suboptimal outcome appears in cases on nonunion [25, 26]. Malunion or shortening of the clavicle under nonoperative treatment may lead to a change of shoulder function. A shortening >10% affects scapular kinematics [27]. In a long-term period, there will be consequences such as acromioclavicular degeneration, rotator cuff dysfunction and furthermore reduction of force. Therefore, patients with highly displaced fractures, resulting in a functional shorter clavicle may benefit by undergoing a surgical procedure [27].

These facts may support a primarily nonoperative management of midshaft clavicle fractures in most cases. However, the challenge is the identification of patients who might benefit from surgical fixation. Patients with persisting pain or a delayed course under conservative treatment may be candidates for early secondary surgery.

### 1.3 Practical Considerations and Techniques of Nonoperative Management

In general, a consequent and strict immobilization of the clavicle is not possible. Based on the tension forces of the muscles of the shoulder girdle, the frequent changes of position during day and night, and the constant respiratory excursions, there is always some motion in the fractured clavicle [9]. In line with these observations, former techniques like painful closed reduction techniques are neither successful regarding enduring alignment nor recommended anymore.

Initial treatment involves immobilization of the affected shoulder. Among other options, a simple sling or a figure-of-eight brace is commonly used. There is no clear evidence regarding the best technique and the duration of immobilization [16]. A figure-of-eight brace is often thought to prevent or reduce secondary fracture shortening during the time of fracture healing. Stepwise tightening of the brace is recommended to counteract the shortening forces. However, there is no evidence for this view and studies have shown no difference between a sling and a figure-of-eight brace regarding healing time and the rate of nonunion [15]. With no evident advantage compared to a sling, the figure-of-eight brace is associated with more discomfort and pain. Nerve compression with temporary brachial plexus palsies and restriction of venous blood return have been reported in the literature [27].

When a sling is used, immobilisation in internal rotation is usually recommended for 3-4 weeks. Self-mobilisation of the elbow out of the sling is required several times a day to avoid stiffening of the elbow. The range of motion of the shoulder should usually be limited to pendulum excercises for the first 1-2 weeks followed by active movements up to the horizontal plane within the first 6 weeks. Free range of motion is usually allowed after 6 weeks [19]. Weight bearing should be avoided until clinical fracture consolidation. However, all these recommendations are rather based on expert opinions and experience than on clear evidence [16].

Many clinicians allow their patients to begin with isometric physiotherapy and resistance exercises depending on residual pain and discomfort. Sporting activities and work, demanding weight bearing and the use of the arm, are usually suspended until the patient is free of pain with radiographic signs of progressing fracture consolidation, usually after 6-12 weeks [21, 18]. Contact sports should be avoided for 3-4 months [18, 21].

Fracture healing may take more time in nonoperative treatment. In a Canadian multicenter randomized controlled trial, mean time to union was significantly higher for conservative treatment compared to plate fixation (28 vs. 16 weeks) [21]. Regular clinical follow-up examinations including radiographs should be performed to monitor fracture healing. Conservatively treated fractures of the clavicular midshaft usually unite between 18 and 28 weeks after the injury [21, 28]. In case there is no union evident on the radiographs at this point in asymptomatic patients, no more clinical and radiological follow-ups are necessary due to the absence of any therapeutic consequences [29, 30]. In symptomatic patients, conversion to surgery may be considered [19, 22].

## CONCLUSION

There is solid evidence in favour of nonoperative treatment for fractures with a displacement of less than 2cm and remaining contact of the bone fragments. Clear indications for conservative treatment *versus* surgical fixation of displaced midshaft fractures have not finally been established yet, leaving some questions and problems unanswered. Furthermore, there are no evidence-based recommendations concerning the kind and duration of shoulder immobilization with no clear advantage for any treatment modality.

Most fractures are suitable for conservative treatment. The indication for primary surgery should individually be based on the patient’s characteristics and needs. The challenge remains to identify the right patient for the right treatment.

## Figures and Tables

**Fig. (1A):**
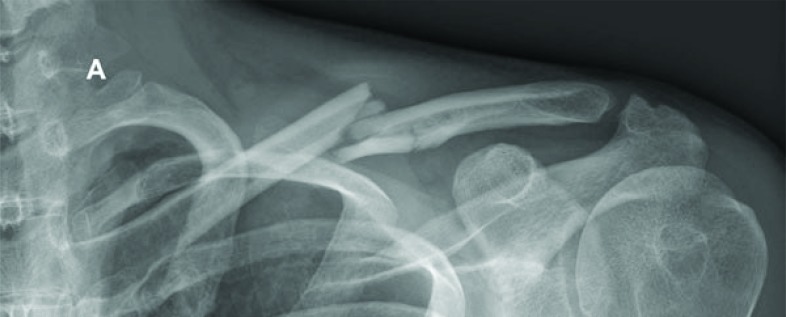
The antero-posterior radiograph shows an acute multifragmentary midshaft fracture of the clavicle with slight displacement and preserved contact of the bone fragments.

**Fig. (1B):**
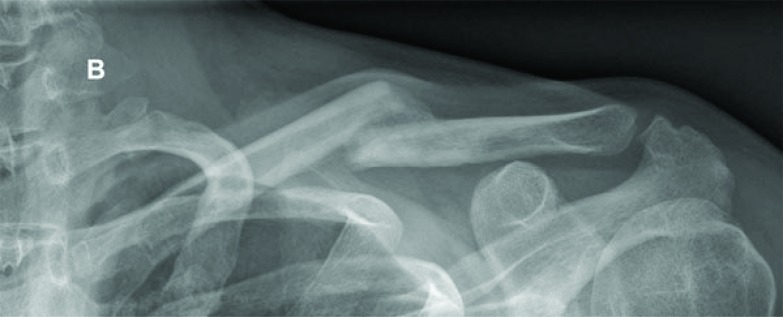
The antero-posterior radiograph of the same fracture at follow-up 16 weeks after trauma demonstrates an only marginal increase of the initial displacement with progressive callus formation.
